# Reconfigurable and Recyclable Photoactuators Based on Azobenzene-Containing Polymers

**DOI:** 10.3389/fchem.2020.00706

**Published:** 2020-08-21

**Authors:** Mingsen Chen, Shuofeng Liang, Chengwei Liu, Yuanli Liu, Si Wu

**Affiliations:** ^1^CAS Key Laboratory of Soft Matter Chemistry, Hefei National Laboratory for Physical Sciences at the Microscale, Department of Polymer Science and Engineering, University of Science and Technology of China, Hefei, China; ^2^College of Materials Science and Engineering, Guilin University of Technology, Guilin, China; ^3^Department of Chemical Engineering, Tsinghua University, Beijing, China; ^4^Max Planck Institute for Polymer Research, Mainz, Germany

**Keywords:** photoresponsive, reprocessable, actuators, polymers, azobenzene

## Abstract

Photoactuators are promising smart materials that can adapt their shapes upon light illumination. Smart materials with recycling, reusable, and reconfigurable properties are crucial for a sustainable society, and it is important to expand their function. Recently, much effort was made to address the issue of reprocessability and recyclability of photoactuators. Based on the development of polymer chemistry, supramolecular chemistry, and dynamic covalent chemistry, it is now possible to prepare reconfigurable and recyclable photoactuators using azobenzene-containing polymers (azopolymers). Herein, the recent advances on reconfigurable and reprocessable photoactuators, including dynamic crosslinked networks systems and non-covalently crosslinked azobenzene-containing polymers, were reviewed. We discuss the challenges in the field as well as the directions for the development of such photoactuators.

## Introduction

Over the past decades, significant progress has been achieved in the development of stimuli-responsive soft materials. They are used for designing soft actuators that show complex, rapid, and reversible macroscopic movements via external or internal stimuli (Hines et al., 2017). Azobenzene-containing polymers (azopolymers) are commonly used to design and fabricate photo-controlled soft actuators (Gelebart et al., 2017b; Lu et al., 2017; Pang et al., 2019). Azopolymers can be switched reversibly between the thermally stable *trans* form and the meta-stable *cis* form because of the reversible photoisomerization of azo bond (Merino, 2011). Ultraviolet (UV) light induces the *trans* azo-form into the *cis* azo-form, and the *cis* azo-form can be converted back to the *trans* azo-form photochemically upon irradiation of visible light or thermally by heating.

Azobenzene mesogens incorporating into liquid crystalline networks (LCN) or liquid crystalline elastomers (LCE) can directly convert light energy into mechanical work (White, 2018). Through rational design of molecular structures and orientations, the soft actuators containing azobenzene moieties can generate sophisticated movements including contraction/expansion (Finkelmann et al., 2001), bending (Yu et al., 2003), oscillation (Kumar et al., 2016), and twisting (Iamsaard et al., 2014), which brings broad applications in artificial muscles, microrobots, microfluidics, and so on. However, the existence of covalently crosslinked networks makes them insoluble and unmeltable. Therefore, they cannot be processed by traditional melt-processing and solution-processing methods.

Crosslinking provides materials with mechanical robustness but sacrifices the reprocessing and recycling performance. With the development of the introduction of post-crosslinkable moieties (Yoshino et al., 2010), dynamic/reversible covalent bonds (Chakma and Konkolewicz, 2019), reconfigurable and reprocessable LCEs and LCNs under high temperatures have been prepared (Han et al., 2016; Ube et al., 2016; Yang et al., 2016; Lu et al., 2017; Lahikainen et al., 2018). Furthermore, photoactuators without chemical crosslinking networks achieved a breakthrough in processing performance compared with the traditional crosslinked systems (Choi et al., 2009; Lv et al., 2016; Chen et al., 2020).

Here, we provide a brief overview of recent advances on reconfigurable and recyclable photoactuators based on azopolymers. New types of photoactuators including covalently crosslinked networks and non-covalently crosslinked networks were reviewed. Some challenges in this field were proposed.

## Recent Advances in Photoactuators Based on Azobenzene-Containing Polymers

### Reconfigurable or Recyclable Photoactuators With Covalently Crosslinked Networks

**Figure 1:**
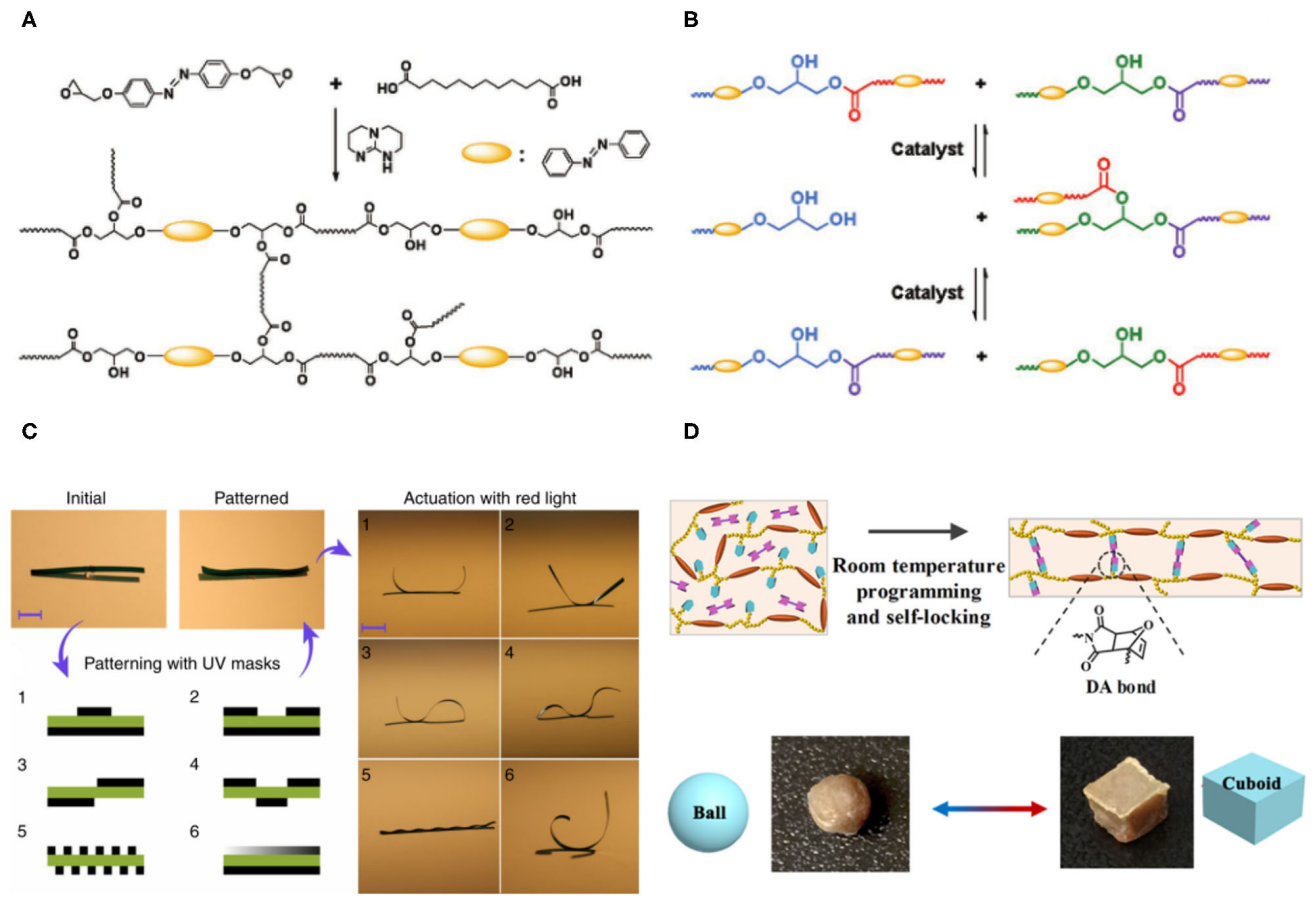
**(A)** Synthesis route of the azobenzene liquid crystalline elastomers. **(B)** Schematic illustration of the reversible transesterification reaction. **(A,B)** Reprinted with permission from Lu et al. (2017) Copyright Wiley-VCH. **(C)** Reconfigurable shape morphing of a single actuator through synergistic use of photochemical and photothermal effects. Reprinted with permission from Lahikainen et al. (2018) Copyright Springer Nature. **(D)** Schematic showing the “self-lockable” liquid crystalline Diels-Alder dynamic network (LCDAN) actuators that can be (re) shaped into 3D objects at room temperature. Reprinted with permission from Jiang et al. (2020) Copyright Wiley-VCH.

Dynamic/reversible covalent bonds can fast break and reform between several molecules under appropriate conditions (Chakma and Konkolewicz, 2019). The introduction of dynamic bonds into photoactuators affords them reprocessability and reshaping ability. Lu et al. (2017) demonstrated that large-size polymer photoactuators can be reconfigured into wheels or spring-like “motors” using a reprocessable azobenzene-containing liquid crystalline network (Figure 1A). The dynamic reaction occurs between an ester bond and a hydroxyl group within the polymer backbone, while it has to react at elevated temperature and catalysts (Figure 1B). At elevated temperature, the catalytic transesterification reaction occurs and allows the epoxy-acid-derived network rearranged without changing the numbers of links and average functionality, and thus affording malleable and easy processing properties (Lu et al., 2017).

Some reconfigurable actuators based on conventional crosslinked networks were developed by synergistic use of photochemical and photothermal effects or interplay between light and humidity or pH (Gelebart et al., 2017a; Lahikainen et al., 2018; Wani et al., 2019). Lahikainen et al. (2018) prepared a reconfigurable actuator that can be programmed to adapt different shapes under an identical stimulus through synergistic use of photochemical and photothermal effects. The basic idea is to use azobenzene photoisomerization to locally control the *cis*-isomer content and to program the actuator response. Afterward, photothermal stimulus was used to actuate the shape deformation of the actuators. Six different shapes reconfigured from one single actuator were demonstrated under identical irradiation conditions (Figure 1C). An initial actuator was irradiated through masked UV exposure from either one side or both sides of the sample to achieve spatially patterning areas with high *cis*-isomer content. The UV pre-irradiation altered the *cis*-content within the actuator. Upon red-light illumination, the actuator quickly deformed into different geometries determined by the UV pre-irradiation (Lahikainen et al., 2018).

Besides, many other reactions promoting exchangeable covalent bonds have also been applied to prepare reprocessable actuators, including transcarbamoylation (Wen et al., 2018), boronic-ester exchange reaction (Saed et al., 2020), and photo-exchange reaction of disulfide (McBride et al., 2018) and allyl sulfide (McBride et al., 2017). All these examples require an external force at high temperatures or under illumination. Developing actuators capable of reprocessing at room temperature can open up large-scale applications. Recently, Jiang et al. (2020) demonstrated a “self-lockable” liquid crystalline Diels-Alder dynamic network actuator that exhibited room temperature programmability and solution-reprocessability. The liquid crystalline dynamic networks can be reprogrammed and self-locked into 3D objects at ambient temperature simultaneously stabilized by slowly formed Diels-Alder bonded crosslinks (Figure 1D). The actuator showed a reversible shape change upon heating above and cooling below the ordered-disordered phase transition temperature. Moreover, such polymers can fabricate a light-fueled walker or wheel (Jiang et al., 2020). This new actuator displayed unmatched recycling and reprocessing performance. It can be dissolved in organic solvents and reprocessed from solution, which cannot be achieved with liquid crystalline networks using exchangeable covalent bonds.

### Reconfigurable and Recyclable Photoactuators Using Non-covalently Crosslinked Networks

Another strategy to produce the recyclable photoactuators is directly using linear photoresponsive materials or supramolecular non-covalent interactions (e.g., hydrogen bonding or other weak interactions) without chemical crosslinking networks. The hydrogen bonding-induced physically crosslinked networks in photoactuators based on azopolymers or hydrogels were reprogrammable or recyclable (Mamiya et al., 2008; He et al., 2009; Ni et al., 2016; Jeon et al., 2017; Nie et al., 2017; Si et al., 2018; Mauro, 2019). Lee et al. synthesized the azobenzene-containing linear polymers via acyclic diene metathesis polymerization (Choi et al., 2009; Kim et al., 2013). Fibers or films were prepared by melt-spinning and solution-casting. However, the mechanical properties of the actuators were insufficient due to the low-molecular-weight (*M*_n_ ≈ 10 kg mol^−1^) of the azopolymer. Design and synthesis of linear liquid crystal polymers with robust mechanical properties can address the abovementioned problem.

**Figure 2:**
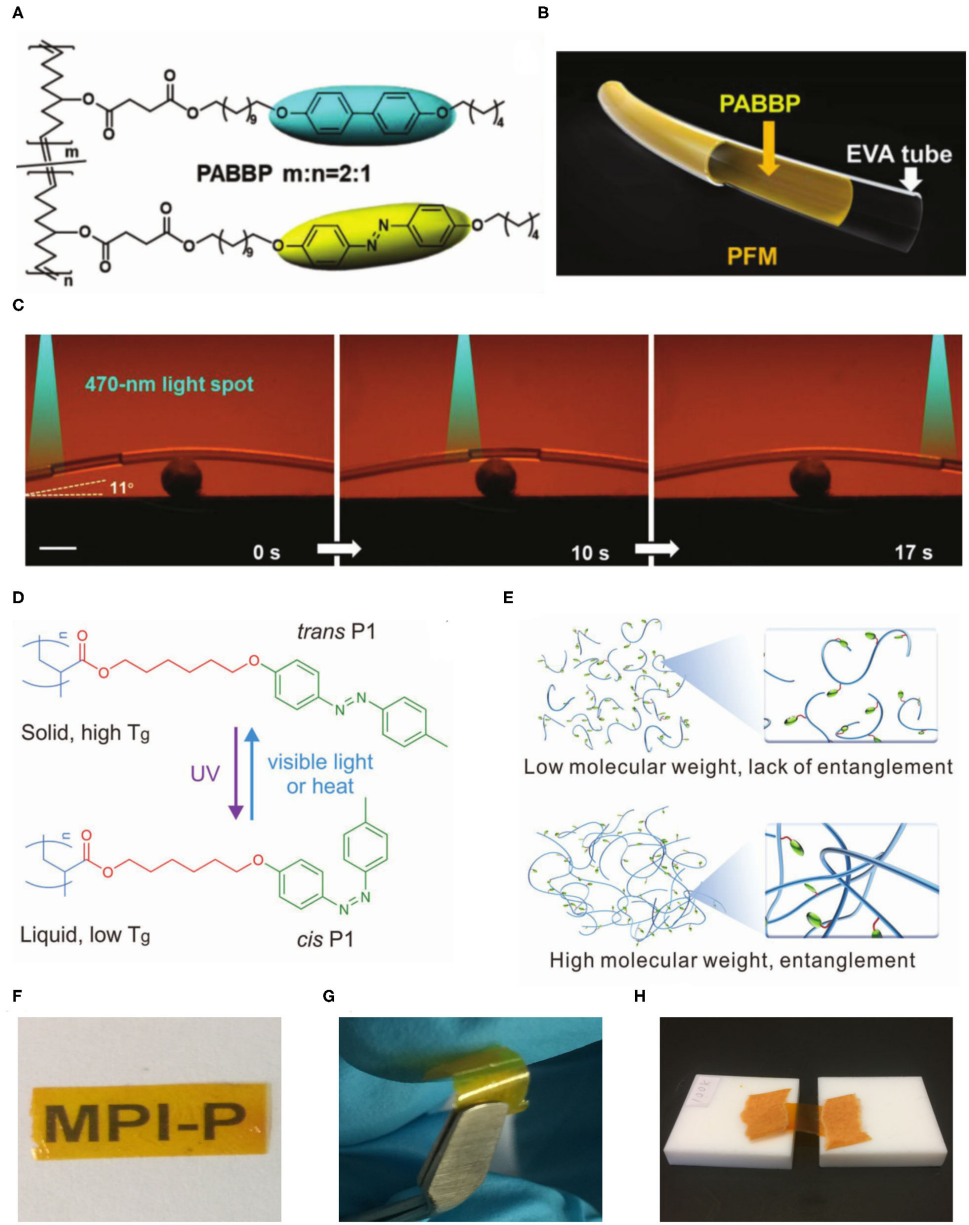
**(A)** Chemical structure of the PABBP copolymer. **(B)** Schematic representation of the bilayer structure of the PFM. **(C)** Photographs of the light-controlled motion of an isopropanol slug climbing over a slope of 11° incline in a curved PFM. The intensity of the 470 nm point light was 120 mW cm^−2^. The scale bar is 2 mm. **(A–C)** Reprinted with permission from Xu et al. (2019) Copyright Wiley-VCH. **(D)** The chemical structure and photoisomerization of P1. **(E)** Schematic representation of entanglement of polymer chains. The freestanding film of entangled P1 was highly transparent **(F)**, flexible **(G)**, and stretchable at 90°C **(H)**. **(D–H)** Reprinted with permission from Chen et al. (2020) Copyright Wiley-VCH.

By imitating the structure of artery walls, Yu and co-workers designed a novel tubular microactuator used for the manipulation of fluid slugs by light (Lv et al., 2016). To improve the mechanical robustness, a high-molecular-weight (*M*_n_ = 360 kg mol^−1^) linear azopolymer was synthesized by ring-opening metathesis polymerization (ROMP) and acted as “photonic muscles” of the microactuator. Subsequently, they further synthesized a linear liquid crystal copolymer (PABBP, *M*_n_ = 300 kg mol^−1^) combining the photoresponsive azobenzenes and biphenyl moieties by ROMP (Figure 2A). By incorporating biphenyl mesogens, light can penetrate deeper in the PABBP layer. Thus, its photodeformability was improved due to the cooperative effect of the two mesogens (Xu et al., 2019). A long bilayer PFM actuator was constructed by the combination of PABBP and the commercially available ethylene-vinyl acetate (EVA) copolymer microtube (Figure 2B). The EVA microtube was selected as the supporting layer due to its good flexibility and comparable modulus. The isopropanol slug confined in PFM was manipulated to climb over a slope of 11° incline by the 470 nm light spot due to photodeformation-induced asymmetric capillary forces (Figure 2C). The PFM actuator was able to reprocess into arbitrary shapes, including knot, helix, and serpentine. Furthermore, the delaminated bilayer PFM can be healed by light due to the photofluidization mechanism of azobenzene moieties, which makes it possible to apply in wearable and integrated microfluidic systems.

Our group synthesized a series of linear liquid crystal azopolymers (P1) with different molecular weights by atom transfer radical polymerization (ATRP). High-molecular-weight entangled azopolymers were able to prepare photoactuators (Chen et al., 2020). These azopolymers showed photoinduced reversible solid-to-liquid transitions. *Trans* azopolymers are solids with glass transition temperature (*T*_g_) values above room temperature, yet *cis* azopolymers are liquids with *T*_g_ values below room temperature (Figure 2D). Photoinduced reversible solid-to-liquid transitions of such azopolymers were applied to light-induced healing and reprocessing of actuators with high spatial resolution. The critical entanglement molecular weight of the azopolymer calculated using Wool's model (Wool, 1993) was 68 kg mol^−1^. The low-molecular-weight azopolymers (5–53 kg mol^−1^) were hard and brittle because their polymer chains lacked entanglements, whereas the high-molecular-weight azopolymers (80–100 kg mol^−1^) entangled (Figure 2E). Thus, transparent (Figure 2F), flexible (Figure 2G), and stretchable (Figure 2H) actuators were fabricated by entangled azopolymers because of mechanical robustness. The photoactuators can be not only recycled and reshaped via solution processing but also reprocessed by light irradiation to produce microstructured actuators (Chen et al., 2020). The combination of polymer chain entanglements and photoinduced reversible solid-to-liquid transitions provides a new strategy for designing actuators with good reprocessability and healability at room temperature.

## Conclusions and Perspectives

In this mini-review, we have summarized the recent progress on reconfigurable and recyclable photoactuators based on azopolymers. The incorporation of dynamic crosslinked networks based on exchangeable covalent bonds, Diels-Alder reactions, or non-covalently crosslinking has been made a breakthrough in the reprocessability of photoactuators. Photoactuators using exchangeable covalent bonds can be processed at high temperatures. Diels-Alder dynamic networks and non-covalently crosslinked networks make actuators recyclable and reprocessable directly from melt or solution processing at room temperature. With the development of improved processing techniques, it provides a new opportunity for designing programmable photoactuators involving the spatially modulation of the orientation of liquid crystals and the control over the geometry and composition. However, so far, the actual applications of photoactuators still face great challenges. In the future, more works should be done to address the following issues:

- Balance material (re) processability, mechanical robustness, and actuating stability: Obviously, crosslinking or entanglement affords mechanical toughness, but it hinders movements of polymer chains, and thus declines reprocessability. Dynamic crosslinked networks provide reprocessability and reconfigurability of the materials. However, during the long-term use of the materials, the dynamic reaction may lead to a reduction in its degree of orientation in actuators, and thus cause performance and reprocessability drops. Constructing new dynamic reaction systems and adjusting the material compositions to achieve a balance between reprocessability, mechanical toughness, and driving stability is of great significance for the practical applications of photoactuators.
- Develop novel non-covalently crosslinked systems and large-scale preparation on demands: The entangled azopolymer photoactuators cannot be large-scale prepared because it is difficult to polymerize high-molecular-weight azopolymers due to the large steric hindrance of azobenzene and other side reactions. In principle, each polymer has a characteristic average molecular weight between entanglements depending on the chemical structure of the repeating unit. This may also be taken into consideration in designing liquid crystal polymers that favor the use of chain entanglements. At present, the synthesis and design of non-covalently crosslinked photoactuators are still in the preliminary stage, and only a few cases are available. Since oriented polymer chains tend to relax in the isotropic state, the actuation degree may decrease after repeated order-disorder phase transition occurs. To exploit new synthetic methods and explore the impact of various chemical structures on their performance will facilitate the large-scale preparation of photoactuators on demand.
- Develop microstructured photoactuators: The recent studies showed that multi-smart functions or properties were accessible using microstructured photoactuators, while obtaining a specific function often required a specifically designed microstructure (Lahikainen et al., 2018; Chen et al., 2020; Ge and Zhao, 2020). Microstructured photoactuators will enable functions such as actuation, detection, transportation, and sensing with potential applications ranging from robotics and photonics to biomedical devices.

## Author Contributions

MC collected and read papers and wrote the manuscript. SL, CL, YL, and SW discussed and revised the manuscript. SW outlined the content of the manuscript. All authors contributed to the article and approved the submitted version.

## Conflict of Interest

The authors declare that the research was conducted in the absence of any commercial or financial relationships that could be construed as a potential conflict of interest.
